# Small Cell Neuroendocrine Carcinoma of the Vagina: A Rare Presentation

**DOI:** 10.7759/cureus.42387

**Published:** 2023-07-24

**Authors:** Asma Asghar, Asma Usman, Ali Zafar Sheikh, Honeyia Imran, Kashif Siddique

**Affiliations:** 1 Radiology/Breast Imaging, Shaukat Khanum Memorial Cancer Hospital and Research Centre, Lahore, PAK; 2 Radiology, Shaukat Khanum Memorial Cancer Hospital and Research Centre, Lahore, PAK

**Keywords:** mri, pet ct, chemotherapy, immunohistochemistry and biopsy, vaginal small cell neuroendocrine carcinoma

## Abstract

Primary small cell neuroendocrine carcinoma of the vagina is a very rare disease. We present a case study of a 52-year-old female who presented to the hospital with complaints of urinary dribbling, burning micturition, pain, and per vaginal bleeding for three to four months. A firm globular mass of approximately 5-6 cm was felt in the anterior vaginal wall per speculum examination. Diagnosis of small cell neuroendocrine carcinoma was made with tissue biopsy and immunohistochemistry. Diagnostic imaging (MRI, positron emission tomography (PET)-CT) plays a vital role in reaching the diagnosis and understanding the treatment response. The patient received six cycles of chemotherapy with cisplatin and etoposide and radiotherapy, achieving a complete response, with complete regression of the lesion. The patient had no sign of tumor recurrence and locoregional or distant metastases after six months of follow-up. Nowadays, there is no need for surgery in the treatment of vaginal small cell neuroendocrine carcinoma, rather radiotherapy and chemotherapy are the treatment of choice. We report a case of neuroendocrine cancer of the vagina treated at our institution.

## Introduction

Only less than 2% of all gynecologic cancer comprises small cell neuroendocrine carcinoma of the vagina [1-3]. It is an extremely malignant aggressive tumor with a poor prognosis [4-6]. It is a very unusual presentation of small-cell neuroendocrine carcinoma as a primary site of the female genital tract. Imaging plays a vital role in the diagnosis of vaginal small cell carcinoma. MRI pelvis shows the exact extent of the disease and its relation with adjacent viscera. Contrast-enhanced CT scan chest, abdomen, and pelvis can help to define the regions of disease involvement and distant metastasis. Recently, PET-CT is the imaging modality of choice, as it shows more accurately the stage of the metabolically avid disease. PET-CT is very supportive in assessing disease regression/progression and reappearance. Chemotherapy and radiotherapy are relatively more effective treatment choices rather than surgery. The key factor regarding this case report is that doctors should be vigilant while dealing with this specific diagnosis so that a patient with non-specific/imprecise symptoms could be assessed and treated in a suitable manner and deliberate diagnosis for a female patient with a vaginal solid mass. Furthermore, the selection of appropriate imaging techniques can further support the diagnosis.

## Case presentation

We present a case of a 52-year-old post-menopausal woman referred to the radiology department with complaints of three to four months related to lower abdominal pain, urinary dribbling, burning micturition, and PV bleeding. She is hypertensive and on medication. Per speculum examination was done, which demonstrates a firm globular mass of approximately 5-6 cm in the anterior vaginal wall, the cervix pushed higher up appeared normal with a small polypoid sub-centimeter mass near os, no sign of a fistula, and normal rectal examination. A transvaginal biopsy was obtained. The immunohistochemistry picture suggested poorly differentiated small cell neuroendocrine carcinoma that revealed malignant neoplasm composed of infiltrative sheets and nests of round hyperchromatic cells with marked atypia and frequent atypical mitotic figures with the markers mentioned below.

Immunohistochemical stain(s):* *Synaptophysin: Positive; p40: Negative; Ki67: 70% proliferation index; CK: Positive; Chromogranin: Negative; CD56: Strong positive; INSM1: Strong positive

**Figure 1:**
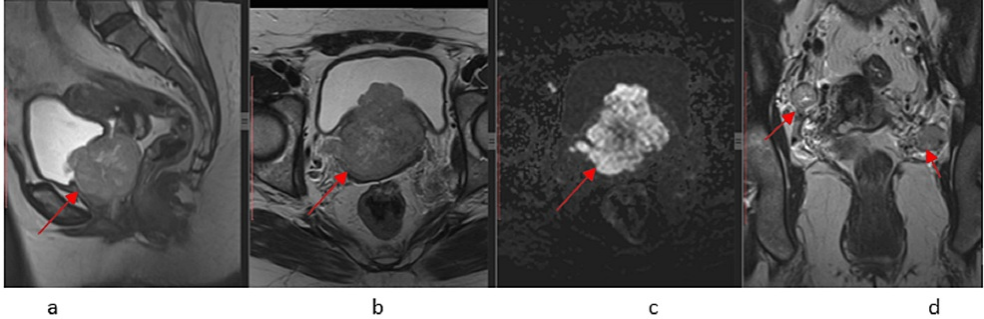
Initial MRI T2 sagittal, axial, and coronal views demonstrate a heterogeneous lobulated mass in the upper two-thirds of the vagina, involving the lower half of the cervix and anteriorly infiltrating into the urinary bladder (1a and 1b), bilateral pelvic side wall lymphadenopathy on coronal T2W1 (1d), and showing restricted diffusion on DWI image (1c) DWI: diffusion-weighted imaging

Imaging studies were done to assess the extent of the disease. A pelvic MRI was performed dated 20.10.2021 that showed heterogeneous signal intensity mass with its epicenter in the upper two-thirds of the vagina, extending superiorly to involve the lower half of the cervix with bilateral pelvic side wall lymphadenopathy. Anteriorly, this mass was infiltrating the urinary bladder through its base and trigone. Posteriorly, it was abutting the rectum without definite infiltration (Figure 1).

**Figure 2:**
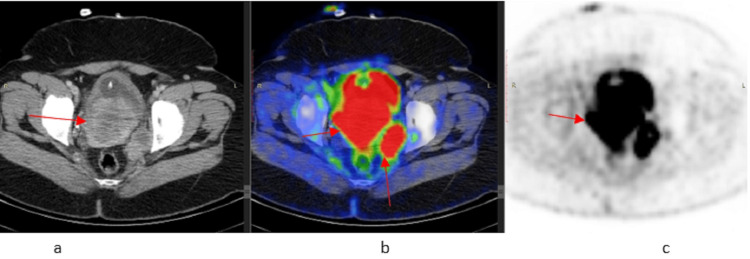
PET-CT demonstrates hypermetabolic mass involving the cervix and vagina with local infiltration of the urinary bladder (2a and 2b) along with hypermetabolic bilateral pelvic side wall lymphadenopathy (2c) PET: positron emission tomography

PET-CT was performed on 6.12.2021 and showed a hypermetabolic tumor involving the cervix and vagina with local infiltration of the urinary bladder consistent with the primary tumor site and hypermetabolic bilateral pelvic sidewall lymphadenopathy. No fluorodeoxyglucose (FDG)-avid nodal or visceral disease was noted elsewhere (Figure 2).

The patient had T4N1Mx (vaginal cervix mass, pelvic sidewall, and bladder infiltration) stage disease on imaging as per the TNM (tumor, node, metastasis) staging system.

**Figure 3:**
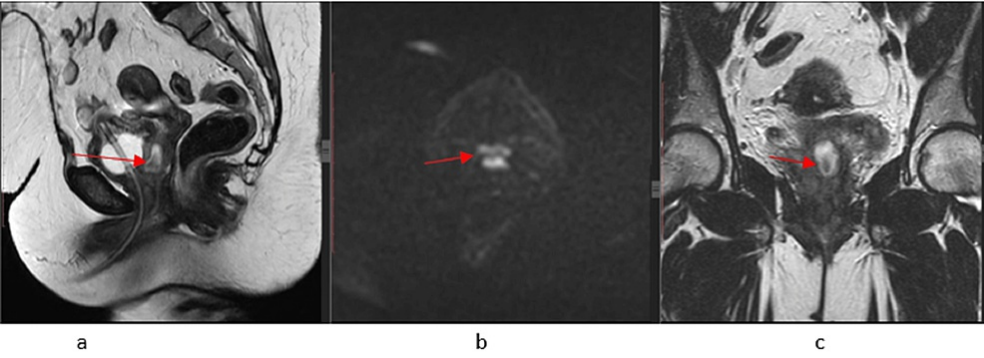
On subsequent MRI while ongoing chemotherapy demonstrates partial treatment response with a significant interval decrease in the size of the soft tissue hyperintense mass involving the upper two-thirds of the vagina as shown in T2 sagittal and coronal images (3a and 3c), which is showing restricted diffusion on DWI (3b); no enlarged pelvic side wall nodes were seen DWI: diffusion-weighted imaging

Interim MRI of the pelvis along with ongoing chemotherapy dated 23.02.2022 showed good partial treatment response in terms of significant interval decrease in size of the soft tissue mass, involving the upper two-thirds of the vagina as well as a decrease in the pelvic side wall lymphadenopathy as described above (Figure 3).

**Figure 4:**
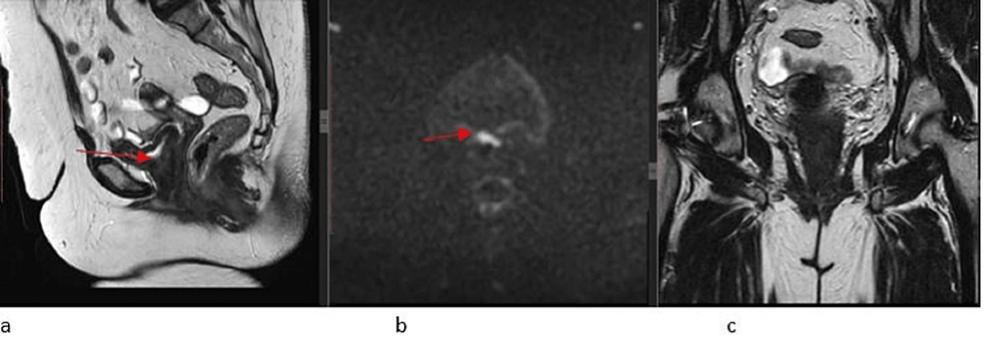
MRI pelvis, post six cycles of chemotherapy shows a good treatment response with a further interval decrease in the abnormal T2 hyperintense signal area involving the upper two-thirds of the vagina on sagittal T2WI as shown by the red arrow (4a), small area of diffusion restriction on DWI (4b) with resolution of pelvic side wall lymphadenopathy (4c); the cervix is clear DWI: diffusion-weighted imaging

Following the six cycles of chemotherapy, an MRI pelvis dated 10.05.2022 showed partial treatment response with further interval decrease in the abnormal signal area involving the upper two-thirds of the vagina with a resolution of pelvic side wall lymphadenopathy (Figure 4).

**Figure 5:**
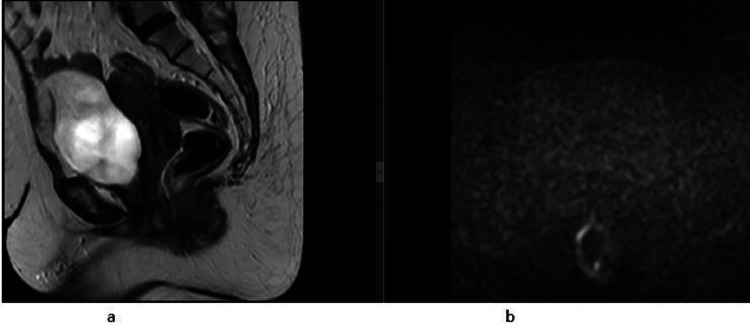
MRI pelvis performed post-chemo-radiation There is interval resolution of previously noted abnormal signal intensity in the upper third of the vagina, the base of the urinary bladder appears grossly unremarkable, and no abnormal diffusion restriction was noted. The pelvic side wall is clear, suggesting a complete response to therapy.

MRI of the pelvis performed (post-chemo-radiation followed by high-dose-rate (HDR) brachytherapy x 4 till 15.07.2022) for response assessment on 10.10.2022 showed a complete radiological interval response with interval resolution of previously noted abnormal signal intensity area in the upper third of the vagina, the base of urinary bladder appeared grossly unremarkable, and no abnormal diffusion restriction was noted (Figure 5).

**Figure 6:**
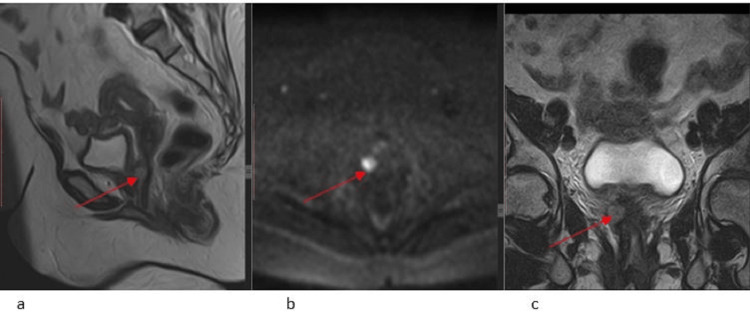
Follow-up MRI pelvis after nine months of completion of therapy demonstrates post-chemoradiation changes with interval development of a circumscribed hyperintense T2 signal area along the right anterolateral aspect of the upper third of the vagina on sagittal T2W1 (6a), corresponding to diffusion restriction on DWI (6b), concerning for recurrent disease DWI: diffusion-weighted imaging

**Figure 7:**
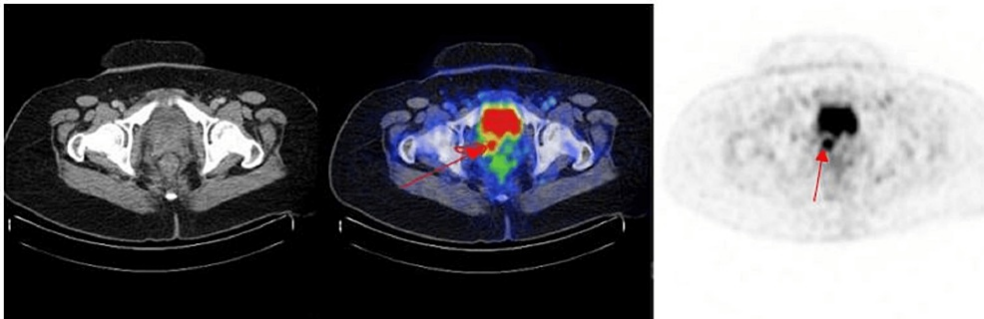
PET-CT shows an FDG-avid nodular lesion along the right anterolateral aspect of the upper third of the vagina concerning for disease recurrence; no FDG-avid disease was seen elsewhere; however, a biopsy of this nodular area showed vaginal tissue with mild chronic inflammation and fibrosis; no in situ or invasive carcinoma was seen PET: positron emission tomography; FDG: fluorodeoxyglucose

Follow-up MRI pelvis and PET-CT were performed on 11.05.2023 and 31.05.2023, respectively, which demonstrate post-chemoradiation changes with interval development of a circumscribed restricting nodule along the right anterolateral aspect of the upper third of the vagina and an FDG-avid nodular lesion in the vaginal stump at its lower aspect consistent with disease recurrence (Figures 6, 7). However, after taking a tissue biopsy, the section shows vaginal tissue showing mild chronic inflammation and fibrosis. No in situ or invasive carcinoma was noted.

## Discussion

Neuroendocrine tumors (NET) occur in a spectrum of malignancies; among them, small-cell cancer is the most aggressive. Often, small cell carcinomas (SmCCs) arise in the lung and only 5% are extrapulmonary [2]. Common in postmenopausal females, the age group for vaginal SmCCs is 38-74 years. In about 2% of malignancies, small cell carcinoma is diagnosed in the female genital tract. Clinical reported features may vary from asymptomatic presentation to dyspareunia, burning micturition, post-menopausal bleeding, and vaginal mass [3,7,8]. The cervix in the female genital tract is the most commonly involved and then, respectively, the ovary, endometrium, vagina, and finally, the vulva.

Being a rare disease, there is no consensus established on treatment yet. The existence of small tumors is restricted to the upper third of the vagina, radical hysterectomy with pelvic lymphadenectomy may be proposed along with partial or complete vaginectomy [8]. Being very rare and despite its aggressiveness, in SmCCs, the chemoradiation regimen controls the local disease. In the case of advanced-stage or non-surgical patients, chemoradiation is the most reasonable treatment option [9-11]. A good survival rate will be considered when appropriate diagnosis and timely treatment was initiated. In our case, adjuvant chemoradiation (etoposide, cisplatin combination chemotherapy with coexisting pelvic radiation therapy, and vaginal brachytherapy) shows excellent treatment response with complete resolution of the disease and no evidence of recurrence seen yet on a follow-up scan.

In 1972, small-cell neuroendocrine carcinoma of the lower female genital tract was first documented by Albores Saavedra et al., as a carcinoid tumor of the uterine cervix [12]. whereas Scully et al. were the first to diagnose, in 1984 small cell carcinoma with the vagina as the primary site [13]. In our patient, the primary site was the upper two-thirds of the vagina, extending up to the lower cervix, with local infiltration into the urinary bladder with pelvic side wall lymphadenopathy; however, at the time of diagnosis, there was no evidence of distant metastasis seen.

Having knowledge of recent imaging techniques will be of significant importance in the diagnosis of vaginal neuroendocrine tumors. In the case of primary vaginal cancer, awareness of MRI features is very useful in diagnosis, local staging, planning of treatment, and complications assessment. Before and after administration of intravenous gadolinium fat-suppressed T1-weighted sequence can be employed to assess tumor enhancement, mainly in evaluating recurrence and/or in patients who have received prior radiation [14]. Fluorine-18 fluorodeoxyglucose-positron emission tomography (18F-FDG-PET), a standard imaging tool for staging and follow-up for vaginal tumors, with improved sensitivity for nodal involvement compared to CT alone [15]. Additionally, for nodal staging and distant disease, CT (simulation with three-dimensional (3D) conformations) is particularly very beneficial for treatment planning and delivery of external beam radiation [14].

## Conclusions

To summarize, primary small cell carcinoma of the vagina is rare and clinically aggressive, with rapid recurrences and distant metastases. Patients should take advantage of the benefits of advanced imaging techniques such as MRI and PET-CT for early diagnosis and prompt commencement/initiating treatment of choice, as the correct diagnosis is important in establishing treatment.
